# Evaluating the Characteristics of the Totally Implantable Venous Access Device in Cancer Patients Using Fluorodeoxyglucose-PET/CT in the Absence of Suspected Catheter-Related Infections

**DOI:** 10.7759/cureus.78621

**Published:** 2025-02-06

**Authors:** Kenichi Kato, Hamano Makoto, Tomohiro Suzuki, Kunihiro Yoshioka

**Affiliations:** 1 Department of Radiology, Iwate Medical University School of Medicine, Shiwa-gun, JPN

**Keywords:** catheter-related complications, fdg pet-ct, malignant tumors, mural thrombus, totally implantable venous access port

## Abstract

Purpose: This study evaluated the fluorodeoxyglucose-positron emission tomography/computed tomography (FDG-PET/CT) imaging features associated with the totally implantable venous access device (TIVAD) in cancer patients.

Methods: We conducted a retrospective single-institution review of TIVAD placements between January 2016 and December 2020. Among these, we identified cases where FDG-PET/CT was performed to monitor malignant tumors after TIVAD placement. Increased accumulation was evaluated based on the site, TIVAD approach, and indwelling time.

Results: In total, 145 TIVAD placements were identified in 144 patients. The median number of FDG-PET/CT examinations was two (range: 1-14). Sites of increased accumulation were found in the vein of catheter penetration (8.3%) and port (1.4%). Increased accumulations were observed only with the subclavian approach. In patients with an indwelling time > 1 month, all accumulations were observed in the vein of catheter penetration (7.9%).

Conclusion: Increased FDG accumulation associated with the TIVAD is not uncommon. A small thrombus with some reactive processes at the vein penetration site may be responsible for the increased accumulation.

## Introduction

Totally implantable venous access device (TIVAD) placement is widely used for administrating intravenous medications in cancer patients. Long-term TIVAD is required for repeated systemic therapy. However, these patients are at risk of catheter-related infection as well as thrombotic complications during long indwelling periods [1-5].

Recently, fluorodeoxyglucose-positron emission tomography/computed tomography (FDG-PET/CT) has been a useful imaging modality for the detection of infectious foci. Miceli et al. reported that FDG-PET is a safe and accurate tool for diagnosing infection of an implantable catheter or port, including those patients who did not exhibit local signs of infection [6]. In addition to infections of implantable vascular devices, Compelman et al. described that FDG-PET/CT seems helpful in differentiating between septic and non-septic thrombosis in patients with catheter-related bloodstream infections [7]. Furthermore, the survival of catheter-related bloodstream infections seems to be improved using FDG-PET/CT in the diagnostic workup [8,9]. Thus, FDG-PET/CT plays an important role in patient management, treatment strategies, and patient outcomes in patients with implantable vascular catheter-related infections.

Although FDG uptake is sensitive to inflammatory and tumor cells [10], the FDG-PET/CT imaging features associated with TIVAD remain unclear in asymptomatic patients. Such excellent functional imaging might detect some potential abnormality related to the management of patients indwelling TIVAD. This study aims to evaluate the imaging characteristics of the TIVAD in cancer patients using FDG-PET/CT conducted during routine follow-up, specifically in the absence of suspected catheter-related infections.

## Materials and methods

Patient selection

This retrospective single-institution study was approved by the Institutional Review Board of Iwate Medical University (approval no. MH2024-106). The institutional radiology database was used to screen consecutive patients. The inclusion criteria were as follows: (i) TIVAD placement in our department between January 2016 and December 2020, (ii) TIVAD implantation for chemotherapy, and (iii) FDG-PET/CT planned as a routine clinical follow-up to evaluate the cancer status after TIVAD placement. The exclusion criterion was having an FDG-PET/CT obtained at another institution. The demographic and clinical data of eligible patients and concomitant imaging studies were collected and evaluated.

TIVAD procedure

All TIVAD procedures were performed by or under the supervision of board-certified interventional radiologists. Central venous catheterization was performed via the subclavian, internal jugular, or cubital vein approach. Central venous catheterization for the subclavian or internal jugular veins was performed under ultrasound guidance. A subcutaneous pocket was created in the anterior upper chest wall except for the cubital vein approach. Fluoroscopic guidance was used to confirm the positions of the catheter and port. All TIVADs were implanted using the 5 Fr open-end polyurethane catheters (TORAY P-U Celsiteport®, Tokyo, Japan).

PET/CT acquisition and interpretation

Patients underwent FDG-PET/CT to evaluate tumor staging or treatment response during clinical follow-up. All PET/CT scans were performed using the Discovery PET/CT 600 Motion apparatus (GE Healthcare, Illinois, CH, USA) or Discovery IQ PET/CT (GE Healthcare) approximately 60 minutes after intravenous injection of 2 MBq/kg to 5 MBq/kg of FDG. Maximum intensity projection (MIP) images and fused FDG-PET/CT images were reviewed using a viewer system (EV Insite.R®, PSP Corp., Tokyo, Japan). All PET/CT studies were interpreted by two diagnostic radiologists (K.K. and M.H., with 26 and six years of diagnostic radiology experience, respectively) with access to the clinical information and imaging reports, and abnormal findings associated with the indwelling catheter or port were reviewed. Any discrepancies in the interpretation of data were resolved in a later discussion. Circular regions of interest (ROI) were set for the increased accumulations to measure the maximum standardized uptake value (SUVmax) on FDG-PET/CT images.

Definition and outcome measures

The observation period was defined as the duration from TIVAD placement to the last FDG-PET/CT scan. Indwelling time was defined as the time from TIVAD placement to the first abnormal finding on FDG-PET/CT or the last FDG-PET/CT in each case. The primary endpoint of this study was the incidence of increased TIVAD accumulation on FDG-PET/CT. The approaches to TIVAD placement and the site of increased TIVAD accumulation were also examined among patients with increased TIVAD accumulation. It was presumed that cases with an indwelling period of less than one month were affected by postoperative accumulation. Therefore, based on the indwelling time, cases with increased accumulation were subdivided into two groups: under or over one month. If available, the secondary endpoint was the changes of increased accumulation in a series of other FDG-PET/CT scans. Furthermore, increased accumulation was compared with contrast-enhanced (CE)-CT, which was limited to one month before and after FDG-PET/CT to reduce the impact of morphological changes.

Statistical analysis

Statistical analyses were performed using the Statcel4 software (OMS Publishing Inc., Saitama, Japan). The Mann-Whitney U test was used to evaluate the differences in continuous variables between the two groups. Statistical significance was set at p < 0.05.

## Results

Patient characteristics

This study identified 144 patients who underwent FDG-PET/CT following TIVAD placement, as retrieved from our database during the study period. In total, 145 TIVAD placements were performed on 144 patients. Table 1 summarizes the patient characteristics. The subclavian approach was chosen for the access route of TIVAD placement in our department; hence, the subclavian approach was used in most cases. Among various malignant tumors, FDG-PET/CT is preferred for hematological disorders; consequently, lymphoma or leukemia is predominant over other disorders. The median observation period was 294 (range: one to 2424) days, and the median number of FDG-PET/CT examinations per TIVAD placement was two (range: one to 14).

**Table 1:** Baseline characteristics of 144 patients (145 TIVAD placements) TIVAD: Totally implantable venous access device

Characteristics	Value
Gender (male/female)	76/68
Age (years, median)	17-85 (60)
Access route	
Subclavian approach	115 (79.3%)
Jugular approach	28 (19.3%)
Cubital approach	2 (1.4%)
Clinical diagnosis	
Lymphoma, Leukemia	87 (60.4%)
Head and neck cancer	14 (9.7%)
Testis tumor	9 (6.3%)
Myeloma	4 (2.8%)
Breast cancer	3 (2.1%)
Lung cancer	2 (1.4%)
Others	25 (17.4%)
Observation period (days, median)	1-2424 (294)
PET examination numbers per TIVAD (median)	1-4 (2)

FDG accumulation after TIVAD placement

Among the 145 TIVAD placements, increased FDG accumulation was observed in 14 (9.7%) (Table 2). All increased accumulations were noted using the subclavian approach, and no increase in accumulation using the jugular or cubital approach was observed. Sites of increased accumulation were found at the vein of catheter penetration or port. To exclude postoperative FDG accumulation, all cases were divided into two groups based on indwelling time. In the indwelling time less than one month group, increased accumulation was observed in three out of five cases (60%). The accumulation was likely a postoperative change, including in two cases of increased port accumulation. In contrast, the indwelling time for the more than one-month group showed increased accumulation in 11 out of 140 cases (7.9%), and all accumulations were observed at the subclavian vein of catheter penetration. No significant difference in indwelling time concerning the presence or absence of increased FDG accumulation was found in the more than one-month group (Table 3).

**Table 2:** Increased FDG accumulation after TIVAD placement FDG: Fluorodeoxyglucose, TIVAD: Totally implantable venous access device

Outcome	Value
Incidence of increased FDG accumulation of TIVAD	14/145 (9.7%)
SUV_max_ of increased FDG accumulation of TIVAD	1.78-4.03 (median 2.38)
Increased accumulation based on the TIVAD approach	
Subclavian approach	14/115 (12.1%)
Jugular approach	0/28 (0%)
Cubital approach	0/2 (0%)
Site of increased accumulation	
Vein of catheter penetration	12/145 (8.3%)
Port	2/145 (1.4%)
Increased accumulation based on indwelling time	
Indwelling time <1 month	3/5 (60%)
Indwelling time >1 month	11/140 (7.9%)

**Table 3:** Comparison between the presence and absence of increased accumulation at indwelling times >1 month

Presence of increased accumulation	Absence of increased accumulation	p-value
11/140 (7.9%)	129/140 (92.1%)	
93-980 days (median 532)	32-2290 days (median 288)	0.35

Changes of increased accumulation

**Figure 1:**
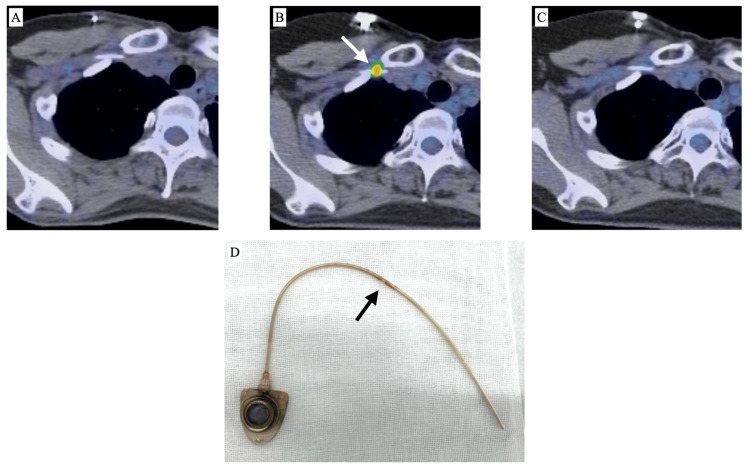
FDG-PET/CT scans and TIVAD of a 40-year-old woman with malignant lymphoma A:  FDG-PET/CT 159 days after TIVAD implantation shows no abnormal accumulation; B: Increased accumulation observed at the catheter penetration site into the subclavian vein (arrow) 560 days after TIVAD implantation; C: Increased accumulation disappeared on FDG-PET/CT 929 days after TIVAD implantation; D: The TIVAD was removed at the end of chemotherapy. A small amount of thrombus-like deposit is observed corresponding to the catheter penetration site (arrow). FDG: Fluorodeoxyglucose, TIVAD: Totally implantable venous access device

**Figure 2:**
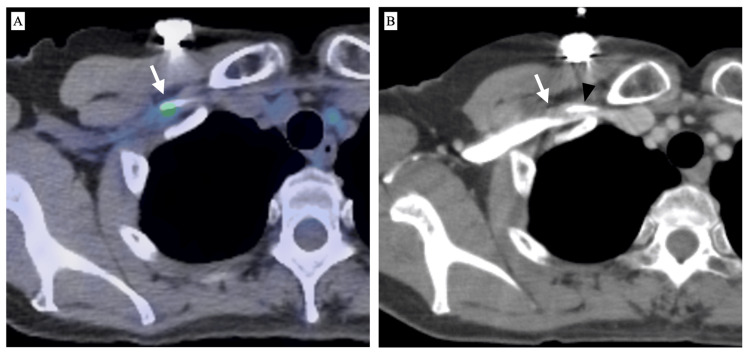
FDG-PET/CT and CE-CT scans of a 59-year-old woman with malignant lymphoma A: FDG-PET/CT 167 days after TIVAD implantation shows a subtle accumulation in the subclavian vein (arrow); B: CE-CT shows a small thrombus (arrow) around the catheter (arrowhead) FDG: Fluorodeoxyglucose, TIVAD: Totally implantable venous access device

Among the 14 cases of increased FDG accumulation, a comparison with a series of other FDG-PET/CT scans was available for 12 cases. Ten cases exhibited a single instance of increased accumulation, while two cases showed two repeated instances of increased accumulation. However, none of the patients showed persistently increased accumulation. Among the 14 cases with increased accumulation, TIVAD removal was performed in one case at the end of chemotherapy. A small amount of thrombus-like deposits was noted corresponding to the catheter penetration site (Figure 1). Comparable CE-CT was available in four cases, and a small thrombus in the subclavian vein around the puncture site was observed in two cases (Figure 2).

## Discussion

This study evaluated the imaging features of the TIVAD using routine clinical follow-up FDG-PET/CT in cancer patients. After excluding cases with an indwelling time of less than one month, which were presumed to reflect postoperative accumulation, increased accumulation of TIVAD was observed in approximately 8% of the catheter-punctured sites in the vein. The interaction between the indwelling catheter and vein has been investigated, and the findings suggest that an indwelling catheter predisposes patients to venous thrombosis [11,12]. Specifically, catheter insertion results in local vessel injury, activating the coagulation and proinflammatory cascades [13].

Catheter-related thrombosis is morphologically divided into four patterns: fibrin sheath, intraluminal clot, mural thrombosis, and venous thrombosis [14]. In our study, increased FDG accumulations were observed around the vessel wall at the access site; they were supposed to represent mural thrombosis containing FDG-incorporated cells. Although a mural thrombus was confirmed in only two cases on available CE-CT, a small thrombus was considered difficult to image. Moreover, no increase in accumulation along the indwelling catheter, suggesting the presence of a fibrin sheath, was observed. Imaging such a very thin layer of the sheath on FDG-PET/CT was presumed to be more challenging compared to the contrast examination of the TIVAD [15].

Histopathologic analysis of the access vein associated with central venous catheters in humans remains limited. Forauer et al. conducted an autopsy study of six patients. In their study, focal areas of catheter attachment to the vein wall consisted of organizing thrombus, collagen, and surface re-endothelialization [16]. Another autopsy study also demonstrated that catheter-related thrombus formation with adjacent inflammatory and fibrotic vessel wall thickening was very common [17]. Based on these autopsy studies, the mechanism of increased FDG accumulation in our study is as follows: local trauma occurring at the catheter’s penetration site into the vein activates thrombus formation with inflammatory and endothelial cell proliferation that traps FDG intracellularly. However, increased FDG accumulations did not persist during follow-up FDG-PET/CT in our study and were observed temporally or intermittently. Additionally, the duration until the occurrence of increased accumulation varied widely. These findings inferred that some reactive processes during catheter penetration into the vein, which cause FDG uptake into the cell, appear randomly. However, the factors that cause this reactive process remain uncertain.

This retrospective single-center study has several limitations. First, our sample was limited, which might have led to a bias in the results. Second, a histopathological correlation corresponding to increased FDG accumulation was not possible. The subtle change in FDG accumulation was speculated to be caused by a reactive process occurring over the mural thrombus. The removed catheter in our case showed small amounts of thrombus-like deposits. However, the pathological analysis of the vein penetration site is not feasible without autopsy specimens. Third, CE-CT at close intervals with FDG-PET/CT was not performed in all cases. Mural thrombosis was observed in only two cases. Moreover, small thrombi are difficult to detect on all imaging modalities, including FDG-PET/CT and CE-CT. Finally, the relationship between the increased accumulation of FDG-PET/CT and clinical significance is uncertain. All enrolled patients underwent FDG-PET/CT for routine clinical evaluation of malignant tumor status, but not aimed at catheter-related infection. No patients had their TIVAD removed based on increased accumulation on FDG-PET/CT. However, the increased accumulation at the vein penetration site might represent the key to the management of catheter-related complications. Larger study groups will be required to clarify these limitations.

## Conclusions

In summary, this retrospective study demonstrates that increased FDG accumulation was observed near catheter penetration into the vein in approximately 8% of the patients with TIVAD placement. The FDG uptake is extremely sensitive to inflammatory lesions, and reactive cells associated with mural thrombus are believed to induce FDG incorporation. These findings are not rare and are not always associated with clinically significant sequelae in terms of monitoring for catheter-related complications. Further studies with larger sample sizes and improved imaging techniques are needed to verify the clinical significance of subtle increases in the FDG accumulation associated with TIVAD.
